# Acute appendicitis before, during, and after the COVID-19 pandemic: a population-based cohort study

**DOI:** 10.3389/fsurg.2026.1919635

**Published:** 2026-09-04

**Authors:** Florin Iordache, Jessica Lazar, Catalin Baraian, Tiberiu Giumba, Roberto Bergamaschi

**Affiliations:** 1 Department of Surgery, Bucharest Emergency Hospital, Bucharest, Romania; 2 Department of Surgery, University of Medicine and Pharmacy “Carol Davila“, Bucharest, Romania; 3 New York Colorectal Cancer Survivorship & Support Group, Jacobi Medical Center, New York, NY, United States

**Keywords:** acute appendicitis, appendectomy, colectomy, laparoscopy, pandemic

## Abstract

**Background:**

Acute appendicitis remains a common surgical emergency. The COVID-19 pandemic severely impacted all the health systems globally including surgical emergencies management. This study aimed to assess changes in diagnosis and treatment of acute appendicitis in a tertiary emergency hospital serving an urban population before, during, and after the COVID-19 years.

**Methods:**

This was a population-based cohort study including consecutive ≥ 18-year-old residents of a metropolitan area presenting with acute appendicitis at the tertiary emergency hospital. The study period between January 2018 and December 2023 was divided into: 2018-2019 (pre-COVID-19); 2020-2021 (COVID-19); 2022-2023 (post-COVID-19). Comparisons among the three periods were performed using one-way ANOVA and the Chi-square or Fisher's exact test as appropriate.

**Results:**

The breakdown of 1,721 patients included was: 662 in 2018-2019, 422 in 2020-2021, and 637 in 2022-2023. Although the catchment area population (1,817 vs. 1,799 vs. 1,781 MM; *p* = 0.2), 27 surgeons (27 vs. 27 vs. 27), and 107,202 CT scans (32,048 vs. 38,806 vs. 36,348; *p* = 0.716) were stable, colonoscopies significantly decreased in 2020-2021 (4,588 vs. 2,457 vs. 4,204; *p* = 0.012). The number of patients presenting with acute appendicitis decreased during the pandemic, with a relative increase in complicated cases. However, complication and mortality rates did not differ. The postoperative length of stay did not change significantly, despite the increased laparoscopic access rates. The appendiceal neoplasm rates were 2.4% in the COVID-19 and 1.9% in the post-COVID-19 year groups.

**Conclusions:**

This six-year population-based study found a decreased number of patients presenting with acute appendicitis during the pandemic years, with a relative increase in complicated cases but no difference in mortality rates. The rates of appendiceal neoplasms raise concerns regarding the offering of conservative treatment.

## Introduction

Acute appendicitis is one of the most common surgical emergency worldwide with a global age-standardized incidence of 214 (174–274) per 100000, corresponding to 17 million new cases and an age-standardized mortality rate of 0.358 (1). The onset of the COVID-19 pandemic in early 2020 posed unprecedented challenges to healthcare systems globally, severely impacting the management of medical conditions. The pandemic necessitated significant shifts in clinical practice to manage the urgent need for surgical care, with the aim of minimizing viral transmission and preserving healthcare resources. These shifts included postponing elective surgery, widely adopting telemedicine, minimizing staff, implementing triage, adapting surgical techniques to minimize length of stay, and reducing training to minimize exposure. In addition, nonoperative treatment was favored by some (2, 3). The measures taken by hospitals had a significant impact on patients. Several studies have demonstrated a reduction in the number of patients admitted for acute appendicitis in hospitals, especially during the first year of the pandemic (4). Others observed that the number of complicated appendicitis was increased during the pandemic (5–7). The explanation provided insisted on the relative increase in the number of patients presenting with complicated appendicitis among all treated appendicitis cases, but also on a delay in hospital presentation (8). Conversely, some studies found no difference between complicated and noncomplicated appendicitis presentations (9). There are also studies suggesting that SARS-CoV-2 could directly interfere with appendicitis, but the evidence is of low quality (10, 11). Moreover, an association between the COVID-19 vaccine and acute appendicitis was studied, but no evidence was found (12, 13). The present study distinguishes itself from previous research by examining extended periods of two years each, preceding the pandemic, throughout the entire pandemic wave, and in the subsequent period. Owing to the extensive data collection conducted in a tertiary emergency hospital, the present study offers a more comprehensive perspective on the changes in the presentation, diagnosis, and management of acute appendicitis before, during, and after the COVID-19 pandemic.

## Method and material

### Study design

This was a retrospective population-based cohort study including consecutive individuals presenting with acute appendicitis to the Bucharest Emergency Hospital. Said patients aged ≥ 18 years resided in the Bucharest metropolitan area up to a 60 km radius corresponding to the catchment area of the Bucharest Emergency Hospital. The defined population was observed longitudinally for a period of time exceeding 6 years. The inclusion of patients started in January 1st, 2018 and was completed in December 31st, 2023. The study period was divided into three 2-year periods: 2018-2019 (pre-COVID-19); 2020-2021 (COVID-19); 2022-2023 (post-COVID-19). The introduction of efficient vaccines in January 2022 changed the progression of the pandemic and constituted the rationale for the selected periods of time. Data were collected for all six years using the hospital's electronic database. This study was conducted in accordance with the Strengthening the Reporting of Observational Studies in Epidemiology (STROBE) guidelines (14).

### Definitions

Acute appendicitis was defined as inflammation of the appendix. The term complicated appendicitis referred to cases involving abscess, gangrene, perforation, or peritonitis (15). Postoperative complications were classified according to Clavien-Dindo (16). Conservative management consisted of keeping the patient *nil per os* (*N*PO) with intravenous (I.V.) crystalloids and intravenous antibiotics (cephalosporin and metronidazole), along with non-opioid analgesics such as paracetamol and ibuprofen. Patients who exhibited clinical deterioration or failed to respond to conservative management underwent surgical procedures.

### Patients

The patients presenting with acute appendicitis were self-referred, sent by family physicians, or transferred from other institutions within the same metropolitan area. All patients underwent a physical examination in the emergency room followed by a comprehensive pre-operative workup including (but not limited to) blood work, EKG, abdominal ultrasound, CT scan, anesthesia consultation and others. Ultrasonography was used as the primary imaging modality because of its availability and rapid accessibility in the emergency setting. Owing to the high volume of emergency admissions, CT scanning was not always immediately available. Once the diagnosis had been established all patients were offered surgery and were asked to sign an informed written consent by the on-call attending general surgeon, who answered the patients' questions to their satisfaction. In case of patients refusing surgery, the attending surgeon offered conservative treatment. No patients who received conservative treatment were managed without prior imaging. atients who underwent conservative treatment received intravenous antibiotic therapy for a minimum of five days, followed by oral antibiotic treatment, resulting in a total treatment duration of at least ten days. The standard i.v. regimen consisted of a cephalosporin combined with metronidazole as per hospital protocol. Patients were not routinely followed after discharge unless they were readmitted. At discharge from the hospital, the patients were referred to the family physicians for follow-up, except for those who were readmitted to the hospital after the initial admission.

### Surgical treatment

Laparoscopic appendectomy was performed according to the technique described in the EAES manual (17). Open appendectomy was performed through a McBurney incision, whereas midline laparotomy was performed in cases of peritonitis and/or septic shock.

### Statistical analysis

Continuous variables were presented as mean and standard deviation (SD) or median and interquartile range for continuous non-normally distributed variables. Categorical variables were presented as frequencies and percentages. Comparisons among the three periods were performed using one-way ANOVA for continuous variables and the Chi-square or Fisher's exact test for categorical variables, as appropriate. A two-tailed *p*-value < 0.05 was considered statistically significant.

## Results

A total of 1721 patients were included in this study. In the “pre-COVID-19” years, there were 662 patients, with 376 (56.8%) male and 286 (43.2%) female patients. In the “COVID-19” years, there were 422 patients, with 245 (58.1%) males and 177 (41.9%) females. In the “post-COVID-19” years, 637 patients were included, with 377 (59.2%) male and 260 (40.8%) female patients (*p* = 0.684). We noted a 36.2% decrease in the number of appendicitis cases from the “pre-COVID-19” period to the “COVID-19” period. An increase of 33.7% from the “COVID-19” period to the “post-COVID-19” period was also noted. The median age of the study group was 36 (range 18-97 years). The most common age range was 30–49 years, compromising 41.3% of patients. This was then followed by 18–29 years (34.4%), 50–69 years (18.0%) and ≥ 70 years (6.4%), showing a significant difference (*p* = 0.025). The baseline demographic data of all patients included in this study are shown in Table 1.

**Table 1:** Demographics, imaging modalities, surgical approaches, complications in the pre-COVID-19, COVID19, and post-COVID-19 periods.

Variable	Total *N* = 1721	Pre-COVID-19 *N* = 662	COVID-19 *N* = 422	Post-COVID-19 *N* = 637	*p* value
Age, median (range)	36 (18-97)	35 (18-97)	35 (18-85)	36 (18-91)	0.440
18–29	592 (34.4)	223 (33.7)	161 (38.2	208 (32.7)	0.162
30–49	710 (41.3)	289 (43.7)	156 (37.0)	265 (41.6)	0.244
50–69	309 (18.0)	115 (17.4)	84 (19.9)	110 (17.3)	0.552
≥ 70	110 (6.4)	35 (5.3)	21 (5.0)	54 (8.5)	0.025*
Sex, *n* (%)					
Male	998 (58.0)	376 (56.8)	245 (58.1)	377 (59.2)	0.684
Female	723 (42.0)	286 (43.2)	177 (41.9)	260 (40.8)	0.684
WBC, *n* (%)					
Leukocytosis	1357 (78.8)	519 (78.4)	343 (81.3)	495 (77.7)	0.803
Normal	351 (20.4)	134 (20.2)	78 (18.5)	139 (21.8)	0.499
Leukopenia	2 (0.1)	1 (0.2)	1 (0.2)	0 (0.0)	1.000
Non-available	11 (0.6)	8 (1.2)	0 (0.0)	3 (0.5)	0.017*
Imaging modality, *n* (%)					
Ultrasound only	1216 (70.7)	489 (73.9)	282 (66.8)	445 (69.9)	0.384
CT scan only	55 (3.2)	15 (2.3)	9 (2.1)	31 (4.7)	0.012*
Ultrasound+CT	329 (19.1)	100 (15.1)	107 (25.4)	122 (19.2)	0.001*
Physical examination only	121 (7.0)	58 (8.8)	24 (5.7)	39 (6.1)	0.097
Surgical approach, *n* (%)					
Open	909 (52.9)	403 (60.9)	231 (61.8)	275 (43.2)	<0.001*
Laparoscopic	755 (43.9)	243 (36.7)	175 (41.5)	337 (52.9)	<0.001*
Conversion	57 (3.3)	16 (2.4)	16 (3.8)	25 (3.9)	0.270
Drainage, *n* (%)					
Yes	1232 (71.6)	455 (68.7)	318 (75.3)	459 (72.1)	0.059
No	489 (28.4)	207 (31.3)	104 (24.6)	178 (27.9)	0.059
Surgical complications, *n* (%)					
Yes	52 (3.0)	7 (1.1)	10 (2.4)	35 (5.5)	<0.001*
No	1669 (97)	655 (98.9)	412 (97.6)	602 (94.5)	<0.001*
Reoperation, *n* (%)					
Yes	19 (1.1)	2 (0.3)	3 (0.7)	14 (2.2)	0.003*
No	1702 (98.9)	660 (99.7)	419 (99.3)	623 (97.8)	0.017*
Clavien-Dindo classification, *n* (%)					
0	1520 (88.3)	553 (85.1)	379 (89.8)	578 (90.7)	0.005*
1	163 (9.5)	95 (14.4)	33 (7.8)	35 (5.5)	<0.001*
2	14 (0.8)	2 (0.3)	6 (1.4)	6 (0.9)	0.121
3	13 (0.8)	2 (0.3)	0 (0.0)	11 (1.7)	0.001*
4	3 (0.2)	0 (0.0)	0 (0.0)	3 (0.5)	0.077
5	8 (0.5)	0 (0.0)	4 (0.9)	4 (0.6)	0.061

*Statistically significant.

Acute appendicitis as a working diagnosis was established in 70.1% of patients in the pre-COVID-19 period, 58.8% in the COVID-19 period, and 61.4% in the post-COVID-19 period, showing a significant difference (*p* < 0.001; Table 2). Leukocytosis was present in the majority of cases but without statistical differences among the three subgroups (Table 1).

**Table 2:** Diagnostic (clinical, pathological), types of appendicitis (uncomplicated, complicated), discharge status, conservative management, and length of stay (LOS).

Variable	Total *N* = 1721	Pre-COVID-19| *N* = 662	COVID-19 *N* = 422	Post-COVID-19 *N* = 637	*p* value
Admission diagnosis, *n* (%)					
Appendicitis	1103 (64.1)	464 (70.1)	248 (58.8)	391 (61.4)	<0.001*
Pain syndromes	470 (27.3)	155 (23.4)	127 (30.1)	188 (29.5)	0.070
Other	148 (8.6)	43 (21.5)	47 (11.1)	58 (9.2)	0.025*
Histology diagnosis, *n* (%)					
Uncomplicated	1174 (68.2)	473 (71.5)	273 (64.7)	428 (67.2)	0.087
Complicated	307 (17.8)	111 (16.8)	89 (21.1)	107 (16.8)	0.041
Negative	143 (8.3)	46 (7.0)	42 (10.0)	55 (8.6)	0.791
Neoplasms	22 (1.3)	2 (0.3)	10 (2.4)	12 (1.9)	0.021*
Incomplete histological report	75 (4.4)	30 (4.5)	8 (1.9)	35 (5.5)	0.001*
Discharge status, *n* (%)					
Alive	1713 (99.5)	662 (100.0)	418 (99.1)	633 (99.4)	0.866
Deceased	8 (0.5)	0 (0.0)	4 (0.9)	4 (0.6)	0.041*
Conservative treatment, *n* (%)					
Yes	26 (1.5)	12 (1.8)	2 (0.5)	16 (2.5)	0.006*
No	1695 (98.5)	650 (98.2)	420 (99.5)	621 (97.5)	0.006*
Length of stay (days, mean ± SD)					
All patients		4.5 ± 2.7	4.6 ± 3.6	4.7 ± 4.8	0.663
Laparoscopic		4.1 ± 2.6	4.4 ± 3.7	4.3 ± 4.4	0.024*
Open		4.6 ± 2.7	4.8 ± 3.5	5.1 ± 5.5	0.701
Conversions		6.2 ± 3.0	6.0 ± 1.4	7.3 ± 2.9	<0.001*
Complicated cases		5.7 ± 3.1	5.6 ± 3.5	6.0 ± 4.7	0.650
Uncomplicated cases		4.2 ± 2.5	4.4 ± 3.5	4.2 ± 3.5	0.968

*Statistically significant.

Radiological investigations (ultrasound and CT scan), whether used alone or in combination, have warranted significant changes over the study period. Ultrasound was the most used imaging modality in the study population, utilized alone in 70.7% of patients. When examined across the three study periods, the observed differences were not statistically significant (*p* = 0.384). However, the “post-COVID-19” period showed a higher use of CT scan (4.7%) in comparison to the other two subgroups (2.3% for “pre-COVID-19” and 2.1% for “COVID-19”; *p* = 0.012). Ultrasound and CT were used more frequently during the COVID-19 period (*p* = 0.001).

In the present study, surgical treatment was offered to all patients diagnosed with acute appendicitis, whereas conservative treatment was limited to patients who refused surgery. The number of surgeons, number of CT-scans and colonoscopies done during the study period and the changes in the catchment area population are depicted in Table 3. Although the laparoscopic approach is generally recommended, open appendectomy was also performed because of various factors, including the unavailability of equipment, the surgeon's experience, case complexity, septic shock, and/or patient preference. Open approach was used more frequently during the “pre-COVID-19” period and “COVID-19 period”, whereas laparoscopy became the main surgical approach in the “post-COVID-19” period (Table 1). A slight increase in the laparoscopic approach was noted throughout the three study periods (*p* < 0.001). Conversion rates remained low, accounting for 3.3% of all cases during the 6-year study period, without statistical differences between subgroups (*p* = 0.270).

**Table 3:** Catchment area population (Bucharest Metropolitan area), number of surgeons, number of CT-scans and colonoscopies done during the study period.

Changes in population, staff and investigations	Pre-COVID-19	COVID-19	Post-COVID-19	*p* value
Catchment area (population of Bucharest)	1.817 mil	1.799 mil	1.781 mil	0.2
Surgeons, no.	27	27	27	-
Colonoscopies, no.	4588	2457	4204	0.012
CT scan, no.	32048	38806	36348	0.716

Conservative treatment was used in 1.5% of our overall population, with 1.8% conservatively treated in the “pre-COVID-19” period, 0.5% in the “COVID-19” period and 2.5% in the “post-COVID-19” period (*p* = 0.006). There was a relative increase in the number of complicated appendicitis cases during the COVID-19 period (Table 2). In terms of complications, there were no statistical differences between the three subgroups, except for a slight increase in Clavien-Dindo grade 1 complications in the COVID-19 period, and grade 3 complications in the post-COVID-19 period (Table 1).

The number of neoplasms increased significantly across the three study periods (0.3%, 2.4%, and 1.9%, respectively; *p* = 0.021). The histological types are presented in Table 4 according to the new classification of the American Society of Colon and Rectal Surgeons (ASCRS) (18). No statistical differences in reoperation rates were found among the investigated periods (Table 1). The postoperative length of stay (LOS) was similar between the subgroups.

**Table 4:** Incidental neoplasms found during the study period (demographics, interventions, pathology and results).

Variable	Total *N* = 22	Intervention type	2018 *N* = 1	2019 *N* = 1	2020 *N* = 2	2021 *N* = 6	2022 *N* = 7	2023 *N* = 5	*p*- value
Age, median (range)	48 (24-82)		68 (68)	77 (77)	71 (59-82)	36 (28-64)	41 (27-74)	56 (24-75)	0.137
Age ranges, *n* (%)									
18–29	4 (18.2)		0 (0.0)	0 (0.0)	0 (0.0)	2 (33.3)	1 (14.3)	1 (20.0)	0.688
30–49	8 (26.4)		0 (0.0)	0 (0.0)	0 (0.0)	3 (50.0)	5 (71.4)	0 (0.0)	0.012*
50–69	5 (22.7)		1 (100.0)	0 (0.0)	1 (50.0)	1 (16.7)	0 (0.0)	2 (40.0)	0.353
≥ 70	5 (22.7)		0 (0.0)	1 (100.0)	1 (50.0)	0 (0.0)	1 (14.3)	2 (40.0)	0.181
Sex, *n* (%)									
Male	13 (59.1)		0 (0.0)	0 (0.0)	1 (50.0)	4 (66.7)	5 (71.4)	3 (60.0)	0.617
									
Female	9 (40.9)		1 (100.0)	1 (100.0)	1 (50.0)	2 (33.3)	2 (28.6)	2 (40.0)	0.617
Surgical approach, *n* (%)									
Open	12 (54.5)		1 (100.0)	1 (100.0)	1 (50.0)	4 (66.7)	3 (42.9)	2 (40.0)	0.586
Laparoscopic	8 (36.4)		0 (0.0)	0 (0.0)	1 (50.0)	2 (33.3)	2 (28.6)	3 (60.0)	0.517
Conversion	2 (9.1)		0 (0.0)	0 (0.0)	0 (0.0)	0 (0.0)	2 (28.6)	0 (0.0)	0.109
LOS (days, mean ± SD)	8.0 ± 6.2		4.0 ± 0.0	6.0 ± 0.0	9.5 ± 3.5	6.3 ± 6.3	7.9 ± 2.0	10.6 ± 10.2	0.640
Admission diagnosis, *n* (%)									
Appendicitis	6 (27.3)		0 (0.0)	0 (0.0)	0 (0.0)	3 (50.0)	1 (14.3)	2 (40.0)	0.577
Acute abdominal syndromes	16 (72.7)		1 (100.0)	1 (100.0)	2 (100.0)	3 (50.0)	6 (85.7)	3 (60.0)	0.540
Pathological diagnosis, *n* (%)									
Appendiceal adenocarcinoma	3	Appendectomy	0 (0.0)	1 (33.3)	0 (0.0)	2 (66.7)	0 (0.0)	0 (0.0)	0.073
Appendiceal adenocarcinoma	1	Right colectomy	1 (100.0)	0 (0.0)	0 (0.0)	0 (0.0)	0 (0.0)	0 (0.0)	0.091
Low-grade appendiceal mucinous neoplasms (LAMNs)	11 (50.0)	Appendectomy	0 (0.0)	0 (0.0)	2 (100.0)	1 (16.7)	6 (100.0)	2 (40.0)	0.001*
Appendiceal neuroendocrine tumor (NET)	5 (22.8)	Appendectomy	0 (0.0)	0 (0.0)	0 (0.0)	2 (33.3)	0 (0.0)	3 (60.0)	0.003*
Adenocarcinoma of cecum	1 (4.5)	Right colectomy	0 (0.0)	0 (0.0)	0 (0.0)	1 (16.7)	0 (0.0)	0 (0.0)	0.517
High-grade mucinous carcinoma (pseudomyxoma peritonei)	1 (4.5)	Right colectomy	0 (0.0)	0 (0.0)	0 (0.0)	0 (0.0)	1 (100.0)	0 (0.0)	0.517
Discharge status, *n* (%)									
Alive	21 (95.5)		1 (100.0)	1 (100.0)	2 (100.0)	6 (100.0)	7 (100.0)	4 (80.0)	0.225
Deceased	1 (4.5)		0 (0.0)	0 (0.0)	0 (0.0)	0 (0.0)	0 (0.0)	1 (20.0)	0.225

*Statistically significant.

As expected, the LOS was shorter in the laparoscopic approach group (*p* = 0.024). Conversion was a risk factor for longer LOS (*p* < 0.001).

## Discussions

The COVID-19 pandemic has disrupted the timely diagnosis and treatment critical for preventing the consequences of acute appendicitis, which can lead to perforation, peritonitis, and potentially death.

In the present study, a significant decrease of 44.3% in the number of patients treated for acute appendicitis was observed during the main pandemic period. This could be attributed to a decrease in healthcare-seeking behaviors due to lockdown measures and fear associated with COVID-19 exposure. These findings are in line with similar studies (9, 19, 20). Historically most of the acute appendicitis cases occur under the age of 30 (10, 11). In this study, the most common ages ranged from 30 to 49 years, slightly older confirming the trend observed also by others (21, 22). These discrepancies underline the importance of investigating the shifting demographic patterns of appendicitis over time. Similar to other studies, we found that males were most likely to develop appendicitis (male-to-female ratio of 1:3, 1:3, and 1:4 for each period, respectively). Despite this observed gender disparity, current research does not answer the question of why this occurs.

In terms of admission diagnostics, we observed a statistical difference in the diagnosis of acute appendicitis during the COVID-19 period. Surgeons were probably more reluctant to consider acute appendicitis and had a more elaborate approach to confirming the diagnosis. This correlates significantly with an increase in the use of imaging methods, such as ultrasound and, especially CT scan (Table 1). The use of artificial intelligence in diagnostics and even in predicting postoperative complications in special times (such as the COVID-19 pandemic) could have helped, as recent data suggest (23). Interestingly, the negative appendectomy rate was not statistically different among the three groups. We also found a relative increase in complicated appendicitis cases during the COVID-19 period (*p* < 0.041, Table 2), which was also reported by others (24, 25). The stretching of health system resources and patients' reluctance to present early to the hospital are probably the main causes of this finding.

The COVID-19 pandemic has posed unique challenges to the implementation of laparoscopic appendectomy as the first-line treatment for acute appendicitis because of the airborne transmission of the virus. As a matter of fact, open surgery was suggested at the beginning of the pandemic instead of laparoscopy by many and, also the use of filtration devices to mitigate the risk of transmission which changed the surgical approach for many (26). At the beginning of the pandemic our medical authorities had enforced this issue until the evidence about the transmission risk emerged. Likewise, conservative treatment was used more frequently during the COVID-19 wave (27). There was no statistical difference in terms of the open or laparoscopic approach between the pre-COVID-19 and the COVID-19 periods. Interestingly, a significant statistical increase in the number of laparoscopic appendectomies was found in the post-COVID-19 period compared with the pre-COVID-19 and COVID-19 periods (Table 1). Although logistical aspects may have hindered the use of the laparoscopic approach more often, a trend toward laparoscopy was demonstrated. No statistical differences in terms of surgical complications were found between the three subgroups, except for a slight increase in Clavien-Dindo grade 1 complications in the COVID-19 period and grade 3 complications in the post-COVID-19 period without a clear cause, and consequently, a higher rate of reoperation in the post-COVID-19 period (Table 1) and a higher mortality rate from the start of the pandemic period (Table 2).

Another interesting result of our study is the unexpected neoplasm prevalence (2.4% in the COVID-19 and 1.9% in the post-COVID-19 years), which aligns with more recent data (28). The unexpected rate of 2.4% of neoplasm findings during the COVID-19 period raises concerns regarding the use of conservative treatment.

### Limitations and strengths of the study

Inherently, this report has the weaknesses of a single-center retrospective study, while its strength is to be found in the population-based design, owing to the fact that the tertiary emergency hospital is the only facility caring for a large metropolitan catchment area and has the highest number of patients reported.

## Conclusion

In conclusion, the present six-year population-based study found that fewer patients presented with acute appendicitis during the pandemic years, with a relative increase in complicated cases but no difference in complications and deaths. The rates of appendiceal neoplasms raise concerns regarding the offering of conservative treatment.

## Data Availability

The data analyzed in this study is subject to the following licenses/restrictions: The data cannot be accessed as it contain possible identification numbers for patients. Requests to access these datasets should be directed to florin.iordache@umfcd.ro.
